# Localization and etiological stratification of non-neoplastic small bowel bleeding via CT imaging: a 10-year study

**DOI:** 10.1186/s13244-024-01778-6

**Published:** 2024-08-01

**Authors:** Yuchen Jiang, Yuanqiu Li, Ziman Xiong, John N. Morelli, Yaqi Shen, Xuemei Hu, Daoyu Hu, Zhen Li

**Affiliations:** 1 Department of Radiology, Tongji Hospital, Tongji Medical College, Huazhong University of Science and Technology, Wuhan, China; 2 Department of Radiology, St. John’s Medical Center, Tulsa, OK USA

**Keywords:** Contrast-enhanced CT scan, Diverticular bleeding, Small bowel bleeding

## Abstract

**Objectives:**

The purpose of this study is to assess the diagnostic efficacy of contrast-enhanced CT scans for small bowel bleeding.

**Methods:**

This retrospective study evaluated patients diagnosed with non-neoplastic small intestinal bleeding (including duodenum) who underwent abdominal CT at our institution from December 2013 to March 2023. Patients were categorized into diverticulum and non-diverticulum groups based on the cause of bleeding. Active bleeding was defined on the CT images as extravasation of contrast material in the intestinal lumen during the arterial phase and/or progressive accumulation of contrast material during the venous phase. We have documented the original report (extracted from the medical record system and additional consultation opinions from senior radiologists), including the presence of active bleeding and its potential bleeding location. Furthermore, two radiologists reassessed the CT images, seeking consensus on the diagnosis between them.

**Results:**

The study included 165 patients, predominantly male, with a median age of 30 years. Active bleeding was identified in 48.3% of patients. Notably, all identified bleeding diverticula in the diverticulum group exhibited cul-de-sac termination. Among the identified causes of bleeding, Crohn’s disease was most prevalent (46.7%, *N* of causes = 64). Significant differences were observed in the diagnostic methods between the diverticulum and non-diverticulum groups, with surgery predominantly applied in the diverticulum group, and endoscopy in the non-diverticulum group (*n* = 49 vs *n* = 15, *p* = 0.001). Contrast agent extravasation was significantly higher in the diverticulum group (*n* = 54 vs *n* = 16, *p* = 0.001), and Meckel’s diverticulum cases appearing tubular were significantly higher than in other diverticulum cases (*n* = 25 vs *n* = 3, *p* < 0.001).

**Conclusion:**

CT allows for a higher detection rate of diverticular bleeding, even if asymptomatic, guiding classification into multiple potentially clinically relevant categories.

**Critical relevance statement:**

Contrast-enhanced CT imaging is effective in determining the location and cause of non-neoplastic small bowel bleeding, especially diverticular bleeding. Therefore, the use of enhanced CT should be prioritized in the diagnosis and management of small bowel bleeding.

**Key Points:**

- CT has potential value in the diagnosis of small bowel bleeding.
- CT imaging suggests possible surgical intervention for active bleeding detection.
- CT diagnoses and localizes small bowel bleeding, aiding in treatment and prioritizing in guidelines.

**Graphical Abstract:**

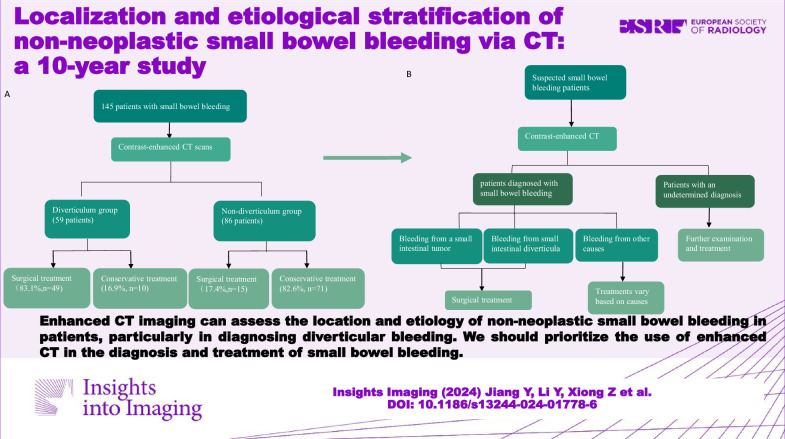

## Introduction

Small bowel bleeding refers to bleeding between the ligament of Treitz and the ileocecal valve, accounting for approximately 10% of gastrointestinal bleeding [1, 2]. In clinical practice, it poses significant diagnostic challenges [3, 4]. Moreover, Chen et al found that diagnosing bleeding in the descending part of the duodenum and its distal segment is challenging and shares similarities with diagnosing small intestinal bleeding [5]. In clinical practice, small intestine bleeding should be broadened to include bleeding from the descending part of the duodenum and its distal segment.

Causes of small bowel bleeding include inflammatory disorders (27.4%), tumors (18.5%), vascular diseases (16.1%), and small bowel diverticula (11.9%) [1, 6, 7]. Chen et al found that intestinal bleeding caused by tumors can be diagnosed and treated via Digital Subtraction Angiography (DSA) [8]. Its diagnostic rate varies from 20% to 77%, influenced by the bleeding rate (bleeding rates: 0.5–1.0 mL/min) [1, 9]. Diverticulosis, inflammatory diseases, and vascular disorders are considered potential non-neoplastic causes of bleeding [6]. Diagnosing non-neoplastic small bowel bleeding is difficult due to subtle and hidden bleeding, complicating endoscopy, angiography, and radionuclide scanning.

Double-balloon enteroscopy (DBE) is the primary diagnostic tool for small bowel bleeding and can also offer therapeutic interventions [10, 11]. However, 15–20% of patients undergoing endoscopic therapy had recurrent bleeding, likely from multiple lesions and limited endoscopic visibility of the entire digestive tract [12, 13]. CT is a valuable adjunct to endoscopic assessment of gastrointestinal bleeding. Contrast-enhanced CT serves two purposes: it locates the bleeding site and potential causes, and provides a detailed view of the small bowel’s anatomy for informed medical decisions [3, 14]. In clinical practice, patients with gastrointestinal bleeding often undergo periods of fasting, abstaining from food and water, as part of their treatment. However, to achieve high-quality enhanced CT images, patients need to consume sufficient fluids to distend the intestines [15]. The priority is given to the stability of a patient’s vital signs over obtaining high-quality images, leading to scans often being performed without complete intestinal distension.

Several studies have highlighted the important role of CT in diagnosing small bowel bleeding and guiding further treatment options [16–18]. Although the detection rate of CT for small bowel bleeding is approximately 40% (95% confidence interval (CI): 33–49%) [1, 19], research has shown that CT can detect lesions missed by endoscopy, such as vascular diseases and inflammatory disorders [20]. The potential of enhanced CT to detect active bleeding has been highlighted in several small-sample studies [18, 21, 22], making it the first-line diagnostic modality for many institutions in diagnosing gastrointestinal bleeding [23, 24]. However, the lack of systematic grouping has hindered clinicians’ clear understanding of its significance. Endoscopy has been considered the primary diagnostic tool in guidelines, with enhanced CT not yet a highly recommended method [1]. Therefore, it is essential to conduct comprehensive and robust analyses and comparisons of relevant cases to provide the necessary evidence for refining clinical diagnostic guidelines.

This retrospective study evaluated the effectiveness of contrast-enhanced CT in diagnosing small bowel bleeding, analyzing all non-neoplastic cases at our institution since 2013, including detailed clinical data and CT images.

## Materials and methods

### Patients

This retrospective study was approved by our institutional review board and the requirement for written informed consent was waived. The electronic medical record system in our institution between December 2013 and March 2023, was reviewed using “small intestinal bleeding” as keywords for searching.

Small bowel bleeding in the present study is bleeding from the descending part of the duodenum to the ileocecal valve. We did not further analyze the causes of intestinal ulcers (such as ulcers caused by nonsteroidal anti-inflammatory drugs, and infections). Inclusion/exclusion criteria were established to select appropriate cases for analysis.

**Fig. 1:**
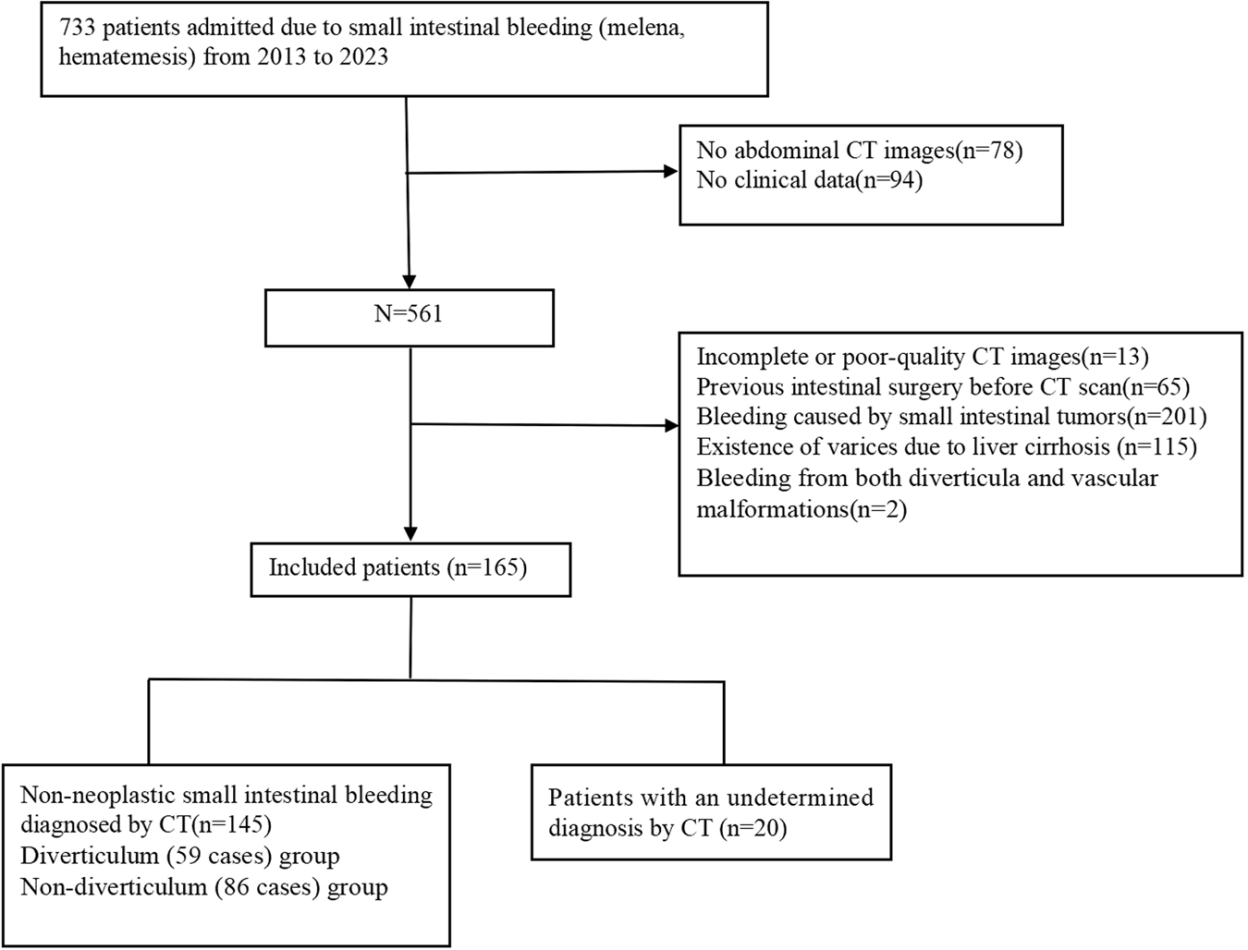
Flowchart for inclusion of patients

Criteria for selecting the subjects were as follows (Fig. 1): (1) patients admitted due to small intestinal bleeding (melena and hematemesis) with no bleeding in other digestive locations (esophagus, stomach, duodenal bulb, and colon) following gastroscopy and colonoscopy, (2) availability of abdominal CT images, and (3) availability of clinical data. Exclusion criteria were: (1) incomplete or poor-quality CT images that could not be analyzed, (2) previous intestinal surgery before CT scan, (3) bleeding caused by small intestinal tumors, (4) presence of varices due to liver cirrhosis, and (5) patients with bleeding from both diverticula and vascular malformations. Clinical data of eligible patients, including age, gender, hemoglobin levels, fecal occult blood test results, examination methods, and treatment approaches were retrospectively reviewed. Compared to bleeding caused by other conditions, diverticular bleeding has a specific characteristic feature: the existence of a cul-de-sac termination [25, 26]. Cul-de-sac termination refers to a closed outpouching that does not have a direct connection to other parts of the intestine. Patients were categorized into either diverticulum or non-diverticulum groups based on the etiology confirmed by surgical, endoscopic, or comprehensive clinical diagnosis. Patients exhibiting bleeding from both diverticular and non-diverticular sources were excluded from both groups in this analysis.

### Image acquisition

All patients underwent abdominal CT in our hospital. The CT equipment was provided by GE Healthcare (Lightspeed 16, Lightspeed 64, Discovery 750, and Brightspeed Elite, Waukesha, WI, USA), Philips Medical Systems (Brilliance 16 and ICT256, Amsterdam, Netherlands), and Toshiba Medical Systems (Aquilion One, Otawara, Japan). The tube voltage of the CT scans was set to 80 kVp/100 kVp/120 kVp, and the automatic control tube current ranged from 110 mAs to 720 mAs. It is recommended to include at least two phases of dynamic contrast-enhanced scans, which meet the CT imaging criteria for inflammatory bowel disease [27].

Due to patients who are acutely ill and cannot tolerate the administration of large volumes of oral neutral contrast agents [1], strict distention of the intestinal lumen is not mandatory in such cases. Hemodynamically stable patients received oral contrast agents, while unstable patients did not. However, the obtained images were still sufficient to meet the diagnostic needs. All patients received intravenous injection of iopromide (Ultraject 370, 370 mg/mL, Bayer Schering Pharma, Berlin, Germany) at a rate of 3–5 mL/s (1 mL/kg or 1.1 mL/kg). Subsequently, a power injector was used to flush with 20 mL of normal saline. Arterial phase images were acquired 30 s after the initiation of contrast material administration, and portal phase images at 60–70 s [14].

### Image interpretation

Active bleeding in CT images was defined as the active extravasation of contrast agents into the intestinal cavity [14, 28]. We recorded the original reports (from the medical record system and additional consultation opinions from senior radiologists), which included the presence of active bleeding and its potential bleeding locations. Additionally, two radiologists re-evaluated the CT images, aware only that the patients were referred for gastrointestinal bleeding, without knowledge of their specific clinical details. In case of disagreement, the radiologists met to reach a consensus agreement. The radiologist evaluation included: (1) the presence of active bleeding; (2) the location of the bleeding: duodenum, jejunum, and ileum; (3) enhancement phase: arterial phase, portal venous phase, and dual-phase; (4) diverticulum shape: tubular (length: base width > 3:1) or pouch-like (length: base width ≤ 3:1) [25]; and (5) distinctive diverticulum indicator: the existence of a cul-de-sac termination.

### Statistical analysis

Statistical analysis was done with SPSS 26.0. Categorical data were analyzed using frequency or percentage, and two radiologists’ agreement was evaluated with weighted Cohen’s kappa. Differences between groups were analyzed using chi-square, Fisher’s exact test, and Student’s *t*-test for normally distributed continuous variables. A *p*-value less than 0.05 (two-tailed) was considered statistically significant.

## Results

The study involved 165 patients, 145 were diagnosed with small bowel bleeding via CT. Among them, 70 patients exhibited active bleeding (48.3%, 70/145). For 75 patients (51.7%, 75/145), CT scans identified only potential bleeding locations. Among 20 cases requiring further diagnosis, 16 were definitively diagnosed via DBE and 4 through surgery. Patients were categorized into diverticulum (59 cases) and non-diverticulum (86 cases) groups for further analysis.

High interobserver consistency between the two radiologists (Cohen’s Kappa = 0.80, 95% CI = 0.72–0.89) was noted in identifying active bleeding. Similarly, a strong consistency was observed between the re-evaluated results and the original reports, yielding a Cohen’s Kappa of 0.72 (95% CI: 0.61–0.84).

### Clinical characteristics of all cases

In this study, we analyzed 165 patients, with 87.9% (145/165) diagnosed with small bowel bleeding through CT. The study population was mainly male, with no significant gender difference between diverticulum and non-diverticulum groups (*p* = 0.07). The study cohort’s median age was 30 years (interquartile range: 16–51 years). Patients in the diverticulum group were notably younger, with a median age of 17 years (range 7–45), compared to 31 years (range 20–48) in the non-diverticulum group (*p* = 0.04). The study’s average initial hemoglobin was 96.57 ± 2.14 g/L, lower in the diverticulum group (84.76 ± 2.42 g/L) than in the non-diverticulum group (99.00 ± 3.26 g/L, *p* < 0.001). Of 30 patients undergoing radionuclide scanning after enhanced CT scanning, 27 had Meckel’s diverticulum. In the study population, 44.2% (*n* = 73) were definitively diagnosed via DBE, 33.9% (*n* = 56) through surgery, 7.3% (*n* = 12) by both surgery and DBE, and 14.6% (*n* = 24) based on comprehensive clinical information. Diagnostic methods varied greatly between groups (*p* < 0.001): surgery predominated in the diverticulum group (67.8%), DBE in the non-diverticulum group (64.0%), and comprehensive clinical diagnosis was more common in the non-diverticulum group (17.4%, 15/86, *p* < 0.05).

**Fig. 2:**
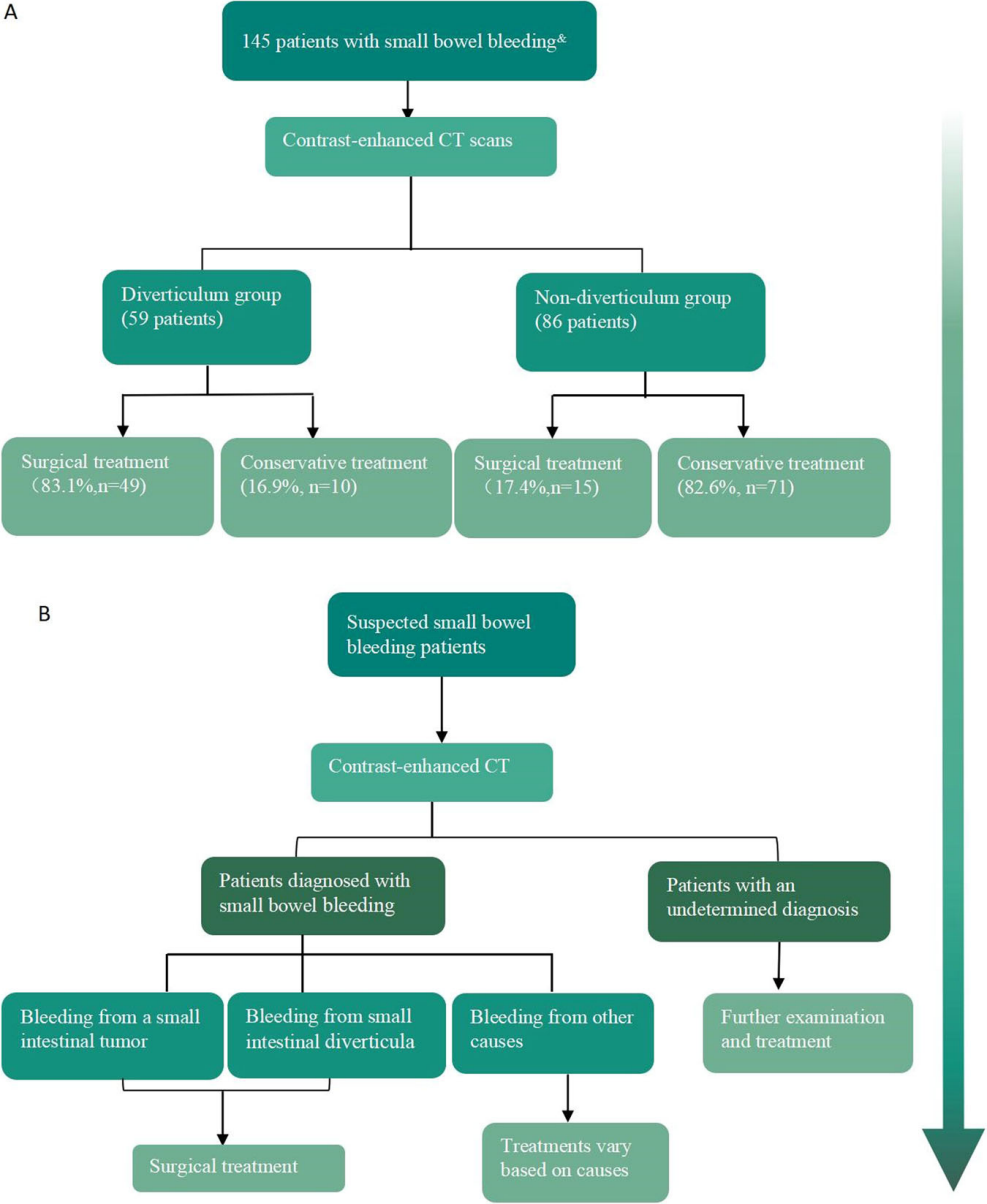
**A** Small bowel bleeding treatment methods. ^&^Bleeding from the descending part of the duodenum to the ileocecal valve. **B** Examination and treatment process for suspected small bowel bleeding patients

The diverticular group had a shorter admission-to-CT interval than the non-diverticular group, though not statistically significant (*p* = 0.16). However, the diverticular group had a shorter time interval from admission to endoscopy compared to the non-diverticular group (*p* = 0.01). Surgical treatment was performed in 41.2% of the patients (*n* = 68), with 33 patients with Meckel’s diverticulum bleeding undergoing surgical intervention. Among 64 patients diagnosed with small intestinal bleeding via CT and treated surgically, 82.8% (53 cases) were accurately diagnosed, 10.9% (seven cases) were misdiagnosed, and 6.3% (four cases) had undetected bleeding sources by CT. Surgical intervention was administered to 83.1% (*n* = 49) of patients within the diverticulum group, in contrast to the predominant employment of conservative management in the non-diverticulum group (Fig. 2A). The recommended sequence of examinations is shown in Fig. 2B. The general characteristics of the study population are summarized in Table 1.

**Table 1:** Clinical characteristics of the study population

Parameters	Total, (*n* = 165) no. (%)	Small intestinal bleeding^d^, (*n* = 145) no. (%)	Diverticulum group, (*n* = 59)	Non-diverticulum group, (*n* = 86)	*p*^e^
Gender					0.07
Male	125 (75.6)	112 (77.2)	50 (84.7)	62 (72.1)	
Female	40 (24.2)	33 (22.8)	9 (15.3)	24 (27.9)	
Age (years)	30 (16, 51)	28 (15, 47.50)	17 (7, 45)	31 (20, 48)	**0.04**
Average initial hemoglobin level (g/L)	96.57 ± 2.14	93.21 ± 2.24	84.76 ± 2.42	99.00 ± 3.26	**0.001**
Positive fecal occult blood tests^a^, (*n* = 135)	98/135 (72.6)	98/122 (80.3)	32/41 (74.1)	66/81 (81.5)	0.65
Diagnosis through other examinations					
Nuclear scanning^b^, (*n* = 30)	19/30 (63.3)	19/30 (63.3)	19/27 (70.4)	0/3 (0)	
DSA^c^, (*n* = 4)	1/4 (25.0)	1/4 (25.0)	0/1 (0)	1/3 (33.3)	
Diagnostic methods					**0.001**
DBE	73 (44.2)	65 (44.8)	10 (16.9)	55 (64.0)	**<** **0.05**
Surgery	56 (33.9)	52 (35.9)	40 (67.8)	12 (14.0)	**<** **0.05**
Both DBE and surgery	12 (7.3)	12 (8.3)	8 (13.6)	4 (4.7)	> 0.05
Comprehensive clinical diagnosis	24 (14.6)	16 (11.0)	1 (1.7)	15 (17.4)	**<** **0.05**
Duration between hospital admission and endoscopy (days)	4 (2.00, 6.00)	3.88 (2.00, 6.00)	3.75 (1.44, 5.91)	4.50 (2.71, 7.00)	**0.01**
Duration between hospital admission and CT (days)	2 (1.00, 3.00)	1.42 (0.92, 2.2)	1.71 (0.96, 2.71)	2.00 (1.00, 3.00)	0.16
Duration between CT and surgery (days)	6 (4.00, 9.75)	6.00 (3.00, 8.00)	6.00 (3.25, 8.00)	8 (5.00, 10.00)	0.06
Treatment					**0.001**
Surgical treatment	68 (41.2)	64 (44.1)	49 (83.1)	15 (17.4)	
Conservative treatment	97 (58.8)	81 (55.9)	10 (16.9)	71 (82.6)	

DSA digital subtraction angiography

Statistically significant *p* < 0.05 values are in bold

^a^ Patients who underwent fecal occult blood test

^b^ Patients whoe underwent nuclear scanning

^c^ Patients who underwent DSA

^d^ Small intstinal bleeding diagnosed by CT

^e^ Comparison between diverticulum group and non-diverticulum group

### Imaging features of small bowel bleeding cases

**Fig. 3:**
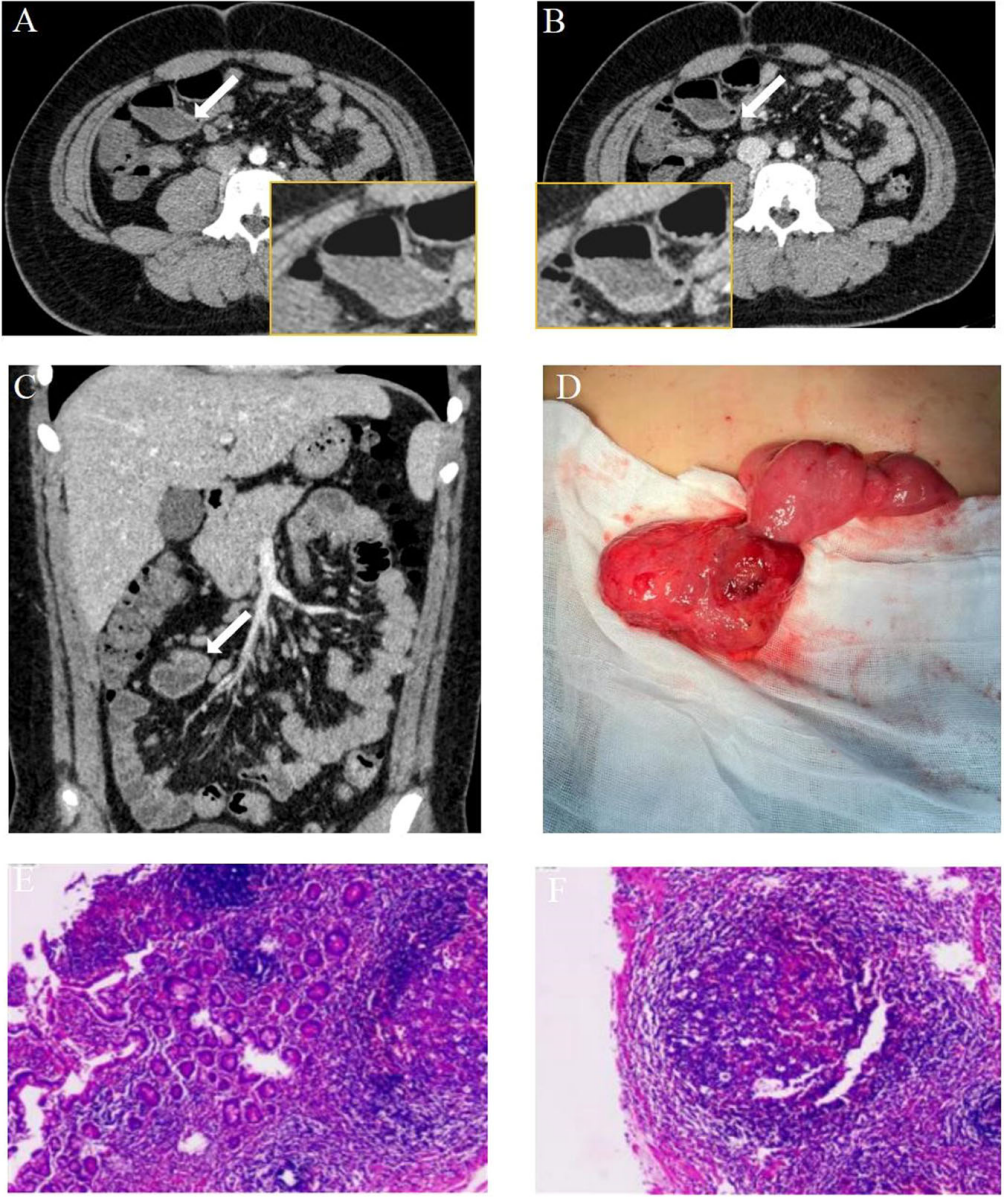
A 12-year-old boy was admitted due to profuse rectal bleeding. One day after admission, an enhanced CT scan was performed. The CT images revealed a sac-like structure with a minimal accumulation of contrast material during the arterial (**A**) and venous (**B**) phases. The CT diagnosis was Meckel’s diverticulum bleeding. **C** Depicts the Meckel’s diverticulum on the coronal plane (white arrow). Ten days later, **D** surgery confirmed the presence of diverticular bleeding located 25 cm from the ileocecal valve, and pathological results (**E**, **F**) indicated Meckel’s diverticulum (HE × 100, × 200)

**Fig. 4:**
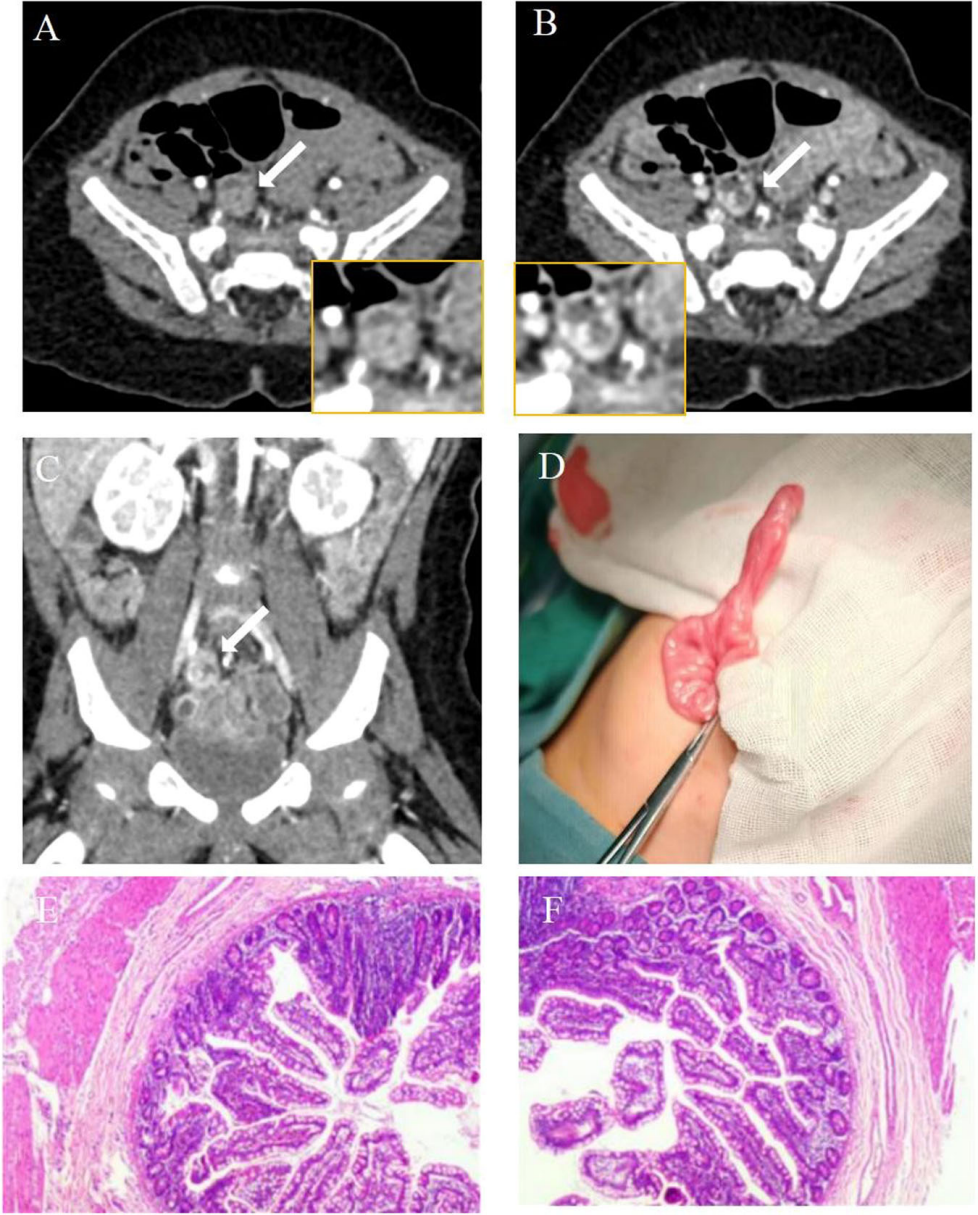
A 6-month-old boy was admitted to the hospital due to recurrent bloody stools, with a hemoglobin level of only 71 g/L. Following an inconclusive diagnosis from an abdominal ultrasound, an enhanced CT scan was subsequently performed, with Meckel’s diverticulum bleeding considered as a preliminary diagnosis. In the arterial (**A**) phase, the image revealed a sac-like diverticulum (as indicated by the arrow), while in the venous phase, the diverticulum gradually filled, observable in axial (**B**) and coronal (**C**) views (as indicated by the arrow). One day after the CT examination, a laparoscopic small intestine resection surgery was performed, and intraoperative images (**D**) showed a diverticular structure in a linear shape (located 35 cm from the ileocecal valve). Subsequent pathological examination (**E**, **F**) confirmed Meckel’s diverticulum (HE × 40, × 40)

**Fig. 5:**
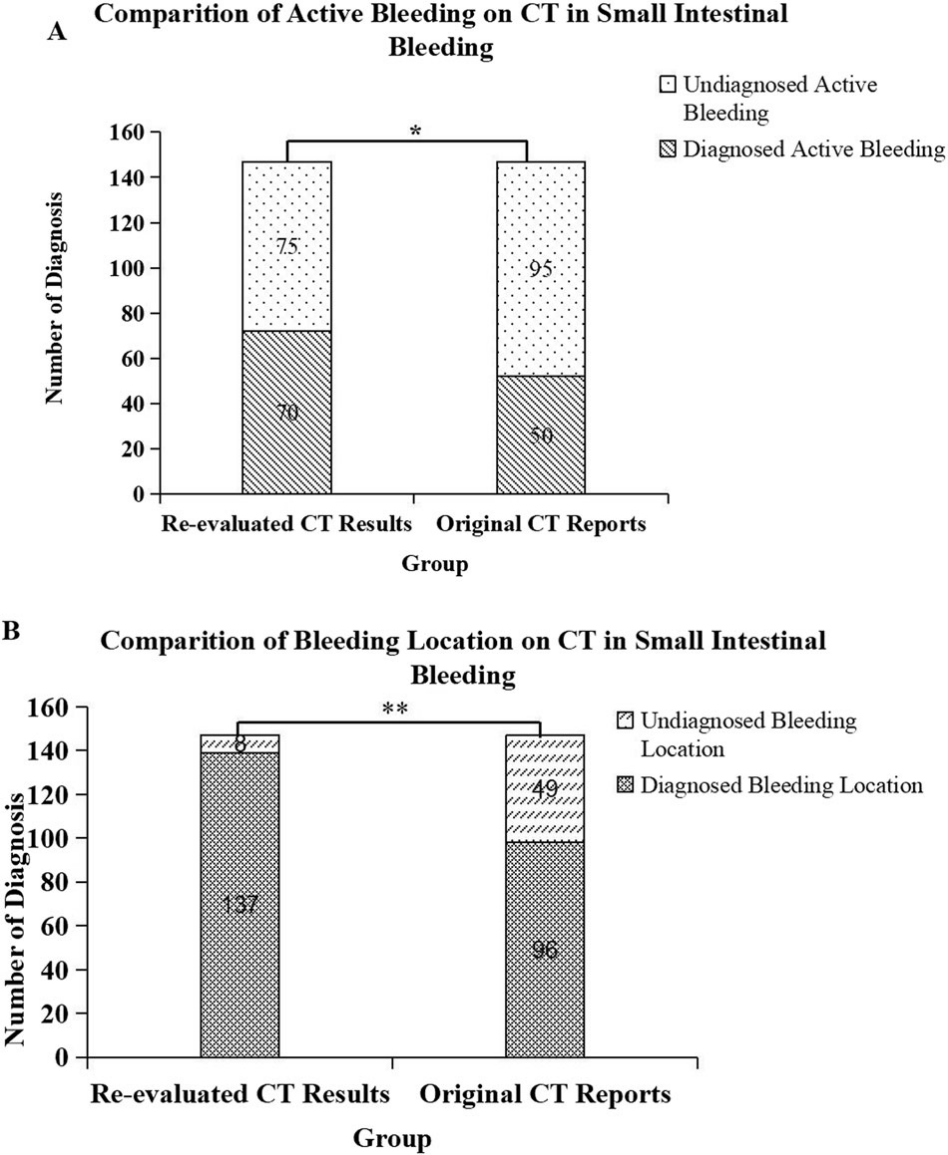
**A** Comparison of active bleeding on CT in small intestinal bleeding. **B** Comparison of bleeding location on CT in small intestinal bleeding. *Indicates a *p*-value less than 0.05, which is statistically significant. **Indicates a *p*-value less than 0.01, which is highly statistically significant

Extravasation indicating active bleeding was observed in 70 patients (48.3%, 70/145, Figs. 3 and 4 and S1 and S2). The vast majority of patients (94.5%, 137/145) displayed potential bleeding sites, totaling 150 locations, all identified through CT examinations. CT diagnosis revealed potential causes of bleeding including Crohn’s disease (46.7%, *N* of causes = 64), Meckel’s diverticulum (23.4%, *N* of causes = 32), ileal diverticulum (8.0%, *N* of causes = 11), vascular malformation (8.0%, *N* of causes = 11), duodenal diverticulum (4.4%, *N* of causes = 6), jejunal diverticulum (4.4%, *N* of causes = 6), hemangioma (2.2%, *N* of causes = 3), radiation enteritis (2.2%, *N* of causes = 3), and jejunal ulcer (0.7%, *N* of causes = 1). Of the total, eight patients presented indeterminate potential bleeding sites and causes on CT. Specifically, three cases were linked to Crohn’s disease, one to jejunal diverticulum, two to Meckel’s diverticulum, one to ileal diverticulum, and one to vascular malformation. Table 2 presents a summary of imaging characteristics for all patients with small bowel bleeding. Re-evaluated CT results indicated a higher rate of active bleeding in small intestinal hemorrhage patients compared to initial reports (48.3%, *n* = 70, vs 34.5%, *n* = 50, *p* = 0.02, Fig. 5A). Additionally, the re-evaluated CT results showed a higher detection rate of potential bleeding lesions than initial reports (94.5%, *N* = 137 vs 66.2%, *N* = 96, *p* < 0.001, Fig. 5B).

**Table 2:** CT characteristics of all small bowel bleeding^a^ patients

CT characteristics	Total, (*n* = 145) no. (%)
Active bleeding^b^	70 (48.3)
Potential bleeding locations^c^ (*N* of lesions = 150)	
Duodenum	11 (7.3)
Jejunum	20 (13.3)
Ileum	119 (79.3)
Potential causes of bleeding^d^ (*N* of causes = 137)	
Duodenal diverticulum	6 (4.4)
Jejunal diverticulum	6 (4.4)
Ileal diverticulum	11 (8.0)
Meckel’s diverticulum	32 (23.4)
Vascular malformation	11 (8.0)
Hemangioma	3 (2.2)
Crohn’s disease	64 (46.7)
Jejunal ulcer	1 (0.7)
Radiation enteritis	3 (2.2)

^a^ Small intestinal bleeding diagnosed by CT

^b^ Active bleeding was visualized on contrast-enhanced CT

^c^ Potential bleeding location obtained from 137 patients where CT could determine the potential bleeding site

^d^ Potential disease sources were identified from 137 patients where CT was able to ascertain the underlying cause of the bleeding

### Comparison of imaging features between the diverticulum and non-diverticulum groups

**Fig. 6:**
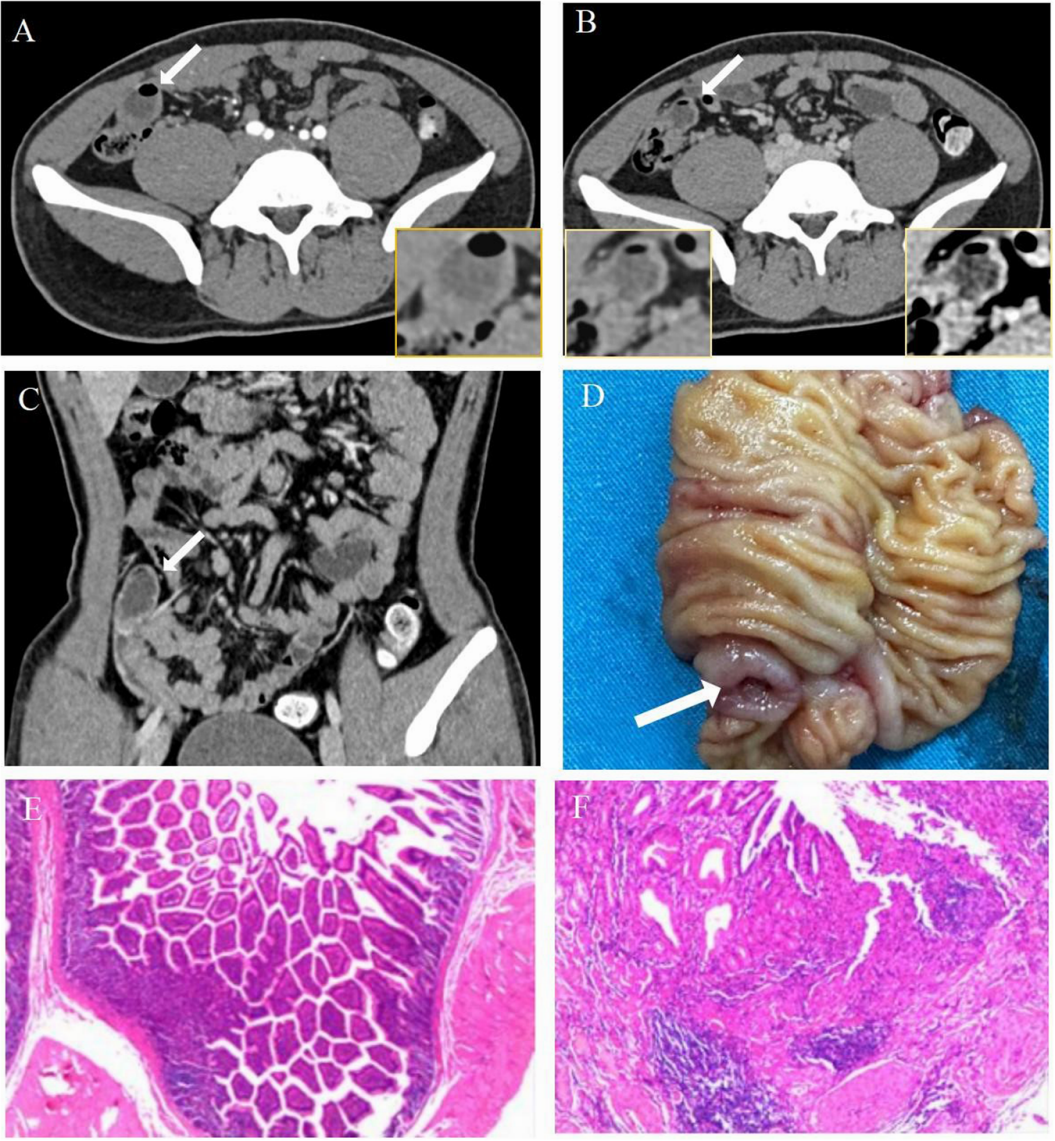
A 19-year-old male patient had been admitted multiple times for melena without a clear diagnosis, and he was once again hospitalized for the same reason. After inconclusive results from a nuclear scan, an enhanced CT scan suggested a diagnosis of ileal diverticular bleeding. In the CT images, during the arterial (**A**) phase, a sac-like diverticulum was visible (indicated by the arrow). The venous phase image (**B**) showed gradual enhancement of the diverticular wall (indicated by the arrow). The coronal view in the venous phase (**C**) revealed a sac-like blind-ended structure. Two days after the CT examination, a DBE was performed (depth of insertion approximately 110 cm from the ileocecal valve), and no abnormalities were found. Ultimately, a laparoscopic partial small intestine resection was performed. Postoperative specimen (**D**) confirmed the presence of diverticular bleeding located 150 cm from the ileocecal valve, but it also revealed intussusception within the diverticulum. Pathological results (**E**, **F**) confirmed ileal diverticular bleeding (HE × 40, × 100)

The proportion of contrast agent extravasation in the diverticulum group was significantly higher than in the non-diverticulum group (91.5%, *n* = 54 vs 18.6%, *n* = 16, *p* = 0.001). Among patients with active bleeding signs on CT, the detection rate of active bleeding through dual-phase images was higher than that of single-phase arterial images, but there was no significant difference between the diverticulum and non-diverticulum groups (*p* = 0.65). Bleeding site identification by CT was slightly higher in the non-diverticular group (95.3%, *n* = 82) compared to the diverticular group (93.2%, *n* = 55), but not statistically significant. The 58 lesions in the diverticular group (identified in 55 patients) all showed cul-de-sac termination (Fig. 6 and S1), with 51.7% (*N* of lesion = 30) presenting as cystic formations and 48.3% (*N* of lesions = 28) appearing as linear shapes on CT scans. The proportion of Meckel’s diverticulum cases appearing tubular was significantly higher than other diverticulum cases (78.1%, *N* of lesions = 25 vs 11.5%, *N* of lesions = 3, *p* < 0.001). Table 3 compares CT features of small bowel bleeding in the diverticulum and non-diverticulum groups.

**Table 3:** Comparison of CT characteristics between the diverticular and non-diverticular groups

Parameters	Diverticulum group, (*n* = 59)	Non-diverticulum group, (*n* = 86)	*p*
Contrast agent extravasation	54 (91.5)	16 (18.6)	**0.001**
Scan phase of contrast extravasation			0.65
Arterial phase contrast extravasation	17 (31.5)	6 (37.5)	
Venous phase contrast extravasation	37 (68.5)	10 (62.5)	
CT-identified bleeding location	55 (93.2)	82 (95.3)	0.86
Potential bleeding location^a^ (*N* of lesions = 150)			0.22
Duodenum	7 (12.1)	4 (4.4)	
Jejunum	7 (12.1)	13 (14.3)	
Ileum	44 (75.8)	75 (81.3)	
Cul-de-sac termination^b^ (*N* = number of lesions)	58		

Statistically significant *p* < 0.05 values are in bold

^a^ Potential bleeding location (*N* = 150) obtained from 137 patients where CT could determine the potential bleeding site

^b^ Cul-de-sac termination was obtained from 60 potential bleeding lesions determined by CT

## Discussion

In this study, small bowel bleeding patients were categorized into diverticulum and non-diverticulum groups. The study compared clinical and imaging findings between the two groups and evaluated treatment strategies. Compared to the non-diverticulum group, the diverticulum group showed a higher proportion of active bleeding on CT images and surgical treatment. Therefore, enhanced CT can guide the stratified management and treatment strategy selection for non-neoplastic small bowel bleeding. Moreover, we found specific signs of diverticula on CT images: cul-de-sac termination, aiding diverticular bleeding diagnosis. Interestingly, compared to other diverticula, Meckel’s diverticulum more frequently exhibits a tubular appearance.

DBE is effective for small bowel bleeding but relies on skilled physicians and patient preparation. Capsule endoscopy is used for non-emergent gastrointestinal bleeding but cannot precisely locate the bleeding source or offer interventional treatment [1]. CT is a convenient and readily accessible imaging technique that is particularly suitable for urgent cases of gastrointestinal bleeding [3]. Moreover, it can help identify the site and etiology of bleeding in hemodynamically stable patients with significant hemorrhage, particularly in assisting intraoperative localization of small bowel diverticular bleeding [4, 14].

In our study, CT images revealed active bleeding in almost half of our patients, and potential bleeding sites were identified through abdominal CT in the majority of patients. However, accurate diagnosis should not rely solely on CT but should also incorporate clinical symptoms and history, particularly in patients with conditions like Crohn’s or prior radiotherapy. The diverticulum group exhibited a higher proportion of active bleeding detected via CT compared to the non-diverticulum group. For these patients, surgical treatment was often the primary approach, whereas conservative therapy was mainly employed for the non-diverticulum group. According to ACG guidelines, surgical intervention is advised for bleeding due to small intestinal tumors or diverticula [1]. In cases of suspected small intestinal bleeding with stable vital signs, an enhanced CT should be the initial step for diagnosis. For other causes, treatment should be cause-specific. If the diagnosis is unclear from the CT scan, further procedures like endoscopy or DSA should be performed to identify the cause before appropriate treatment is given (Fig. 2B). Moreover, we found that patients who exhibit active bleeding on CT images often require surgical intervention, while those who show non-active bleeding on CT images typically undergo conservative treatment. Kennedy et al’s research findings also support this perspective. They discovered that the majority of patients (86%, 19/22) who demonstrated active bleeding on CT images required intervention treatment such as surgery or DSA embolization, or experienced poor prognosis. On the other hand, patients (92%, 59/64) who did not exhibit active bleeding on CT images typically did not require further intervention in a short time interval or until the next onset of bleeding [29]. This highlights the role of contrast-enhanced CT in facilitating stratified management for patients with small bowel bleeding.

Compared to the non-diverticulum group, it is not surprising that the diverticulum group had a younger median age, as the etiology of small bowel bleeding is age-related [30]. Vascular malformation, tumors, and small bowel ulcers are more common in older patients, while younger patients are more likely to have bleeding due to inflammatory bowel disease and small bowel diverticula [1, 14, 30]. This aspect underscores the importance for radiologists to tailor the focus area based on the patient’s age when investigating bleeding. Additionally, the average hemoglobin level in the diverticulum group was lower than that in the non-diverticulum group, indicating that small bowel diverticular bleeding is more occult, difficult to detect, and poses a greater challenge for clinical diagnosis. The diverticulum group mainly confirmed the diagnosis through surgery, while the non-diverticulum group primarily relied on endoscopy for confirmation. The difference can be attributed to two factors: (1) the challenging endoscopic visualization of the diverticula located far from the insertion point; (2) most diverticulum group patients require surgical treatment, while the majority of non-diverticulum group patients can be managed conservatively.

At present, there is a scarcity of research concerning imaging investigations for small bowel bleeding, and even fewer studies that specifically target diverticular bleeding. Kawamoto et al analyzed and compared the detection rate of symptomatic and asymptomatic Meckel’s diverticulum using CT and found a detection rate of 47.5% (19/40) [31]. Despite Nakatsu et al emphasizing the role of contrast-enhanced CT scans in gastrointestinal bleeding, the diagnostic value of CT has been underestimated [32]. The extravasation of the contrast agent was more evident in the venous phase compared to the arterial phase. This may be because the bleeding rate was slow or the amount of bleeding was small, making it less apparent in the arterial phase and only visible in venous phase images. Venous phase images enhance diagnostic confidence in detecting low-flow bleeding. Guglielmo et al also believe that contrast agent extravasation in cases of slow or small-volume bleeding is more commonly observed during the venous phase [14]. Pouw et al proposed that the inclusion of venous phase imaging can enhance the specificity of abdominal CT in the diagnosis of small bowel bleeding [33]. This perspective finds reinforcement in the pig model experiment conducted by Dobritz et al [34]. While it has not been systematically debated, there is no evidence indicating that an extra scanning phase would yield more information. Therefore, when the objective is to tackle issues related to gastrointestinal bleeding, a two-phase enhancement should suffice for clinical stratified management, thereby potentially minimizing the related radiation exposure. Moreover, the marked improvement in detecting active bleeding and potential lesions in small bowel hemorrhage through re-evaluated CT scans (Fig. 5A, B) underscores the importance of second readings in complex diagnostic cases. These findings highlight the benefits of enhanced radiological assessment, suggesting its crucial role in improving diagnostic accuracy in challenging scenarios.

When there is a high suspicion of gastrointestinal bleeding, the primary focus is on stabilizing vital signs [35, 36]. Accurate identification of bleeding sites and causes via CT can be challenging. In non-critical cases, initial enhanced CT scans and improving image quality through adequate intestinal filling may increase CT’s diagnostic efficiency in detecting bleeding.

Studies have shown that 2–25% of small bowel bleeding cases are missed by endoscopy [37, 38]. CT can comprehensively evaluate the gastrointestinal tract and partly compensate for the limitations of endoscopy [3]. The higher CT detection rate of active bleeding in the diverticulum group compared to the non-diverticulum group is likely due to the latter’s association with low-bleed inflammatory diseases, which are less detectable.99mTc red blood cell scan can detect bleeding rates as low as 0.1 mL/min [39]. Dolezal et al conducted a prospective study involving 40 patients and found that the accuracy of red blood cell scintigraphy in detecting small bowel bleeding was 75% [40]. However, Murphy et al reported that red blood cell scanning only provides an approximate location of the bleeding site [30]. The specific nature and management of nuclear medicine examinations make them unsuitable for emergency situations. Moreover, angiography is suitable for large bleeding volumes or fast flow rates in intestinal bleeding [1]. Enhanced CT excels in detecting low-flow bleeding (as low as 0.3 mL/min) and precisely locating it, making it particularly suitable for identifying small-volume, non-tumorous small intestinal bleeding compared to other methods [1].

There are several limitations to our study. First, this study was a retrospective study. Second, we did not analyze pre-contrast images because dual-phase contrast-enhanced imaging is sufficient for diagnosing small bowel bleeding. Pre-contrast images do not provide additional diagnostic value for small bowel bleeding. Third, the high prevalence of positive cases in our cohort may lead to an overestimation of test accuracy.

Our study indicates that CT not only effectively identifies diverticular bleeding but also provides high-definition anatomical details. This assists clinicians in determining subsequent treatment decisions and intraoperative localization, thus being recommended as a priority in clinical guidelines.

### Supplementary information

ELECTRONIC SUPPLEMENTARY MATERIAL

## Data Availability

Data sharing is not applicable to this article as no datasets were generated or analyzed during the current study.

## Abbreviations

DBE: Double-balloon enteroscopy
DSA: Digital subtraction angiography
