# Supplementary material for: Localization and etiological stratification of non-neoplastic small bowel bleeding via CT imaging: a 10-year study
Source: Insights Imaging. 2024 Aug 1;15:189. doi: 10.1186/s13244-024-01778-6 (PMC11294299; doi:10.1186/s13244-024-01778-6)
Supplement: Supplementary file 1 — ELECTRONIC SUPPLEMENTARY MATERIAL [file 13244_2024_1778_MOESM1_ESM.pdf]

Localization and Etiological Stratification of Non-Neoplastic Small Bowel Bleeding via CT: A 10-Year Study
ELECTRONIC SUPPLEMENTARY MATERIAL

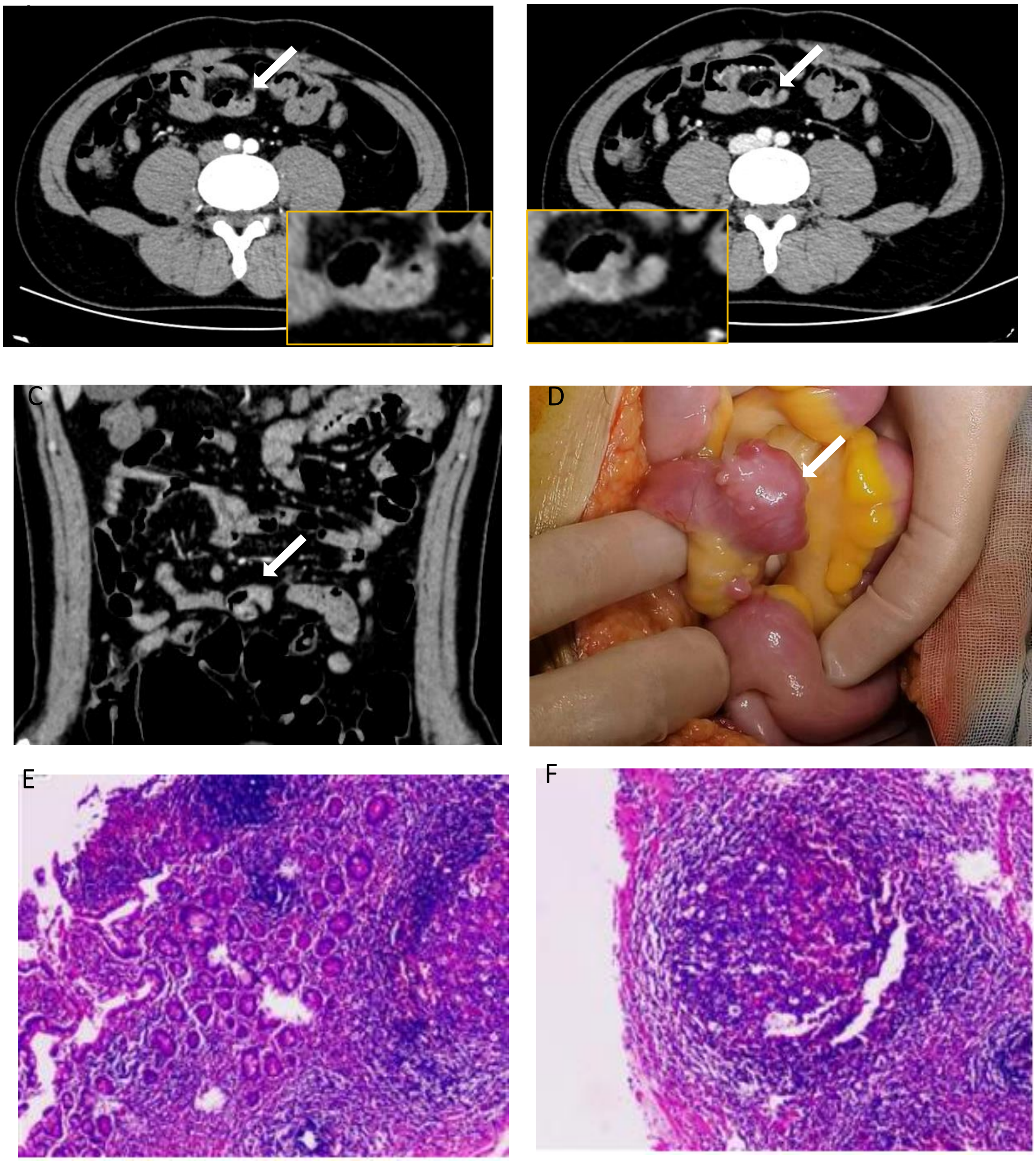

Fig.S1 A 30-year-old male was admitted for rectal bleeding, with an initial hemoglobin level of 54g/L. Following an abdominal contrast-enhanced CT scan, the preliminary diagnosis considered was ileal diverticular bleeding. In the arterial phase (**A**), the CT image revealed the termination of the sac (indicated by the arrow). In the venous phase, axial (**B**) and coronal (**C**) CT images displayed gradual enhancement of the diverticulum (indicated by the arrow). Two days after the abdominal contrast-enhanced CT scan, a follow-up DSA examination showed no abnormalities. Subsequently, a small incision exploratory laparotomy was performed, which revealed bleeding from an ileal diverticulum located 80 cm from the ileocecal valve. The lesion was then surgically removed(D).

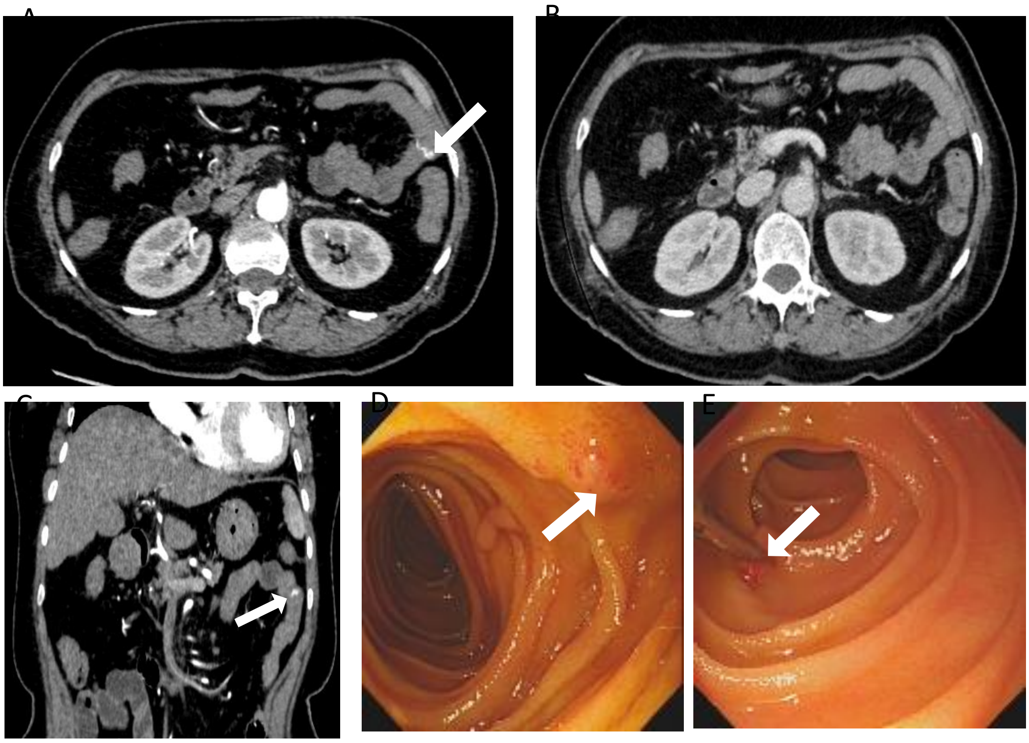

Fig.S2: A 63-year-old woman was admitted multiple times for hematochezia episodes without a clear diagnosis. During this admission, she presented with melena. Enhanced CT scan revealed bleeding from a jejunal vascular malformation. During the arterial phase (A, C), patchy areas of enhancement were observed in the jejunum, highlighted by arrow. In contrast, the venous phase image (B) displayed no such enhancement. Two days after the CT scan, she underwent a double-balloon enteroscopy that reached approximately 150cm past the ligament of Treitz. A bleeding lesion located approximately 100cm past the ligament of Treitz was successfully treated and tagged with a titanium clip(**D-E**). Six days later, a targeted partial resection of the small intestine, using the clip as a landmark, pathologically confirmed a vascular malformation as the source of the bleeding.
